# Associate Psychological Practitioners (APPs) in primary care: modelling the impact

**DOI:** 10.1017/S146342362400032X

**Published:** 2025-01-09

**Authors:** Fiona Lord, Miranda Budd, Kathryn Jane Gardner, Gita Bhutani, Debbie Nixon

**Affiliations:** 1 Workforce Analytics, Planning & Development Manager NHS Lancashire and South Cumbria Integrated Care Board, UK; 2 Pennine Care NHS Foundation Trust (PCFT), Rochdale, UK; 3 School of Psychology, University of Central Lancashire, Preston, Lancashire, UK; 4 Lancashire and South Cumbria NHS Foundation Trust (LSCFT), Preston, Lancashire, UK; 5 Innovation Agency North West Coast, UK

**Keywords:** Associate psychological practitioner, primary care, impact, general practitioner, workforce modelling

## Abstract

**Background:**

The ‘Associate Psychological Practitioner’ (APP) is an innovative new role that expands the psychological workforce and addresses the rising demand for mental health services in England, yet the impact of this role on NHS workforce capacity has yet to be modelled.

**Aim:**

We modelled the impact of the APP role in Primary Care in terms of additional capacity to provide mental health care and the impact on General Practitioner (GP) capacity within the sector.

**Method:**

Workforce experts of the NHS Workforce Repository and Planning Tool (WRaPT) team used a modelling tool to determine future state scenarios of APPs working across all Primary Care Networks (PCNs) within a region and the associated change on the baseline workforce. Modelling was based on Lancashire and South Cumbria, a large geographical area in North-West England that includes 41 PCNs. Assumptions used in the modelling included identifying the patient population and workforce in scope, documenting the activity undertaken by APPs, and considering the future state scenarios for modelling.

**Findings:**

With regard to generating additional capacity, having 1 APP in each of the 41 PCNs in Lancashire and South Cumbria could provide 53 000 brief intervention appointments of 45 min each, thereby diverting these appointments away from the GP, and up to 48 people could benefit from attending Group and Well-being sessions over a year with 1 APP working with another Primary Care colleague, that is, 384 group intervention sessions delivered. In relation to GP capacity, 1 APP (if placed across a PCN, or within multiple practices) could free up at least 1,665 GP appointments within one year, which could lead to potential cost savings. These findings can be used to underpin decision-making with respect to training future cohorts of APPs and contribute to wider workforce planning in primary care.

## Introduction

Early in 2019, the National Health Service (NHS) issued the Long-Term Plan (LTP; (NHS England, 2019) followed by the NHS People Plan in 2020 (NHS, 2020). Both plans recognized the need for an increased supply of an appropriately skilled and motivated workforce, to meet predicted demand and improve outcomes for the population. These included increasing the number of mental health services staff by over 27 000 by 2024 (Health Education England: NHS, 2021). At least one in six people in England report experiencing mental health symptoms (NHS Digital, 2016), with this number expected to have risen further since then, particularly since the COVID-19 pandemic (Office for National Statistics, 2022). As a result, the emphasis on the need for increased workforce supply into the mental health sector has been made in many policy documents over the past five years, e.g., Five Year Forward View for Mental Health (NHS: Mental Health Taskforce, 2016) and Stepping Forward to 2020/21 (Health Education England (HEE), 2017).

Yet, there are workforce challenges across disciplines in the NHS, particularly in the field of mental health, with vacancies standing at 9.7% for all staff working in the mental health sector and 11.8% for nursing in the MH sector (NHS Digital: Q1 2022/23, 2022), and in the NW specifically, vacancies in June 2022 for MH stood at 3,530 (NHS Digital: Vacancy Stats, June 2022). These vacancies, coupled with the rising demand, put significant pressure on services and negatively impact the care users of services receive. Neither the existing nor traditional approaches to increasing the workforce can meet these ambitions. Innovative new roles and new ways of working are required to expand the psychological workforce.

The LTP identifies Primary Care Networks (PCNs) developed in England in 2019 as providing fertile ground for new flexible ways of working across multiple general practices, and in June 2022, NHS England announced funding for 2,500 ‘Mental Health Practitioners’ to provide mental health care, (Baird and Beech, 2020), in primary care settings, to help meet the rising mental health need to increase General Practitioner (GP) capacity to provide routine care (NHS England).

In 2021/22, Mental Health Practitioners were included in the ‘Additional Roles Reimbursement Scheme’ which supports the recruitment of staff into PCNs, paving the way for a new PCN-based psychological workforce. In Lancashire and South Cumbria, there are 41 PCNs, the largest of which has over 90 000 registered patients. Growth of the psychological workforce in these PCNs would improve mental health prevention and promotion, meeting the recommendations set out by the General Practice Forward View (NHS England, 2016), the RCGP (Thomas and Morris, 2016) and (MIND, 2017)

Alongside the rising demand for mental health services is a ‘bottleneck’ of Psychology University graduates who wish to work in the field of psychological healthcare. Psychology is a School that graduates around 29 405 a year nationally in BPS-accredited courses (HESA, 2019/2020) and at least 79% of these wish to pursue a career in mental health care upon graduation (Palmer *et al.*, 2021), but in comparison to other degrees which lead to employment in the NHS, Psychology is an outlier with a huge disparity between career aspirations and an eventual career in mental health (Palmer *et al.*, 2021; Budd, *et al.*, 2022). Upon completion of the Undergraduate degree, there is no immediate graduate entry route for these individuals into the NHS, despite the demand for the skills they can bring within the NHS. Rather, psychology graduates typically spend several more years gaining clinically relevant experience for around three years (National Collaborating Centre for Mental Health, 2019) with the hope of securing a place on a HEE three-year funded Doctorate in Clinical Psychology. This deters many from entering the NHS and only a small proportion of all Psychology graduates (around 1,300; Palmer *et al.*, 2021) eventually become registered Psychologists, with many graduates left to find alternative routes into the NHS that allow them to work psychologically. Moreover, the rise in mental health difficulties in the wake of the COVID-19 pandemic adds further pressure on an already over-stretched NHS and calls for more readily accessible psychological interventions for individuals and local communities following the pandemic and its effects.

Lancashire and South Cumbria is a large geographical area in Northwest England with a mix of city, town and rural communities. The population is around 1.78 million people and is diverse in terms of ethnicity, health and life expectancy, deprivation and wealth, housing and living environment and education and employment, bringing many challenges but also opportunities for the health and care system. Within the area, there are four acute hospital providers, one community/mental health and learning disability provider, four local authorities, and a large independent sector social care provision. The workforce across health and social care equates to over 92 000 whole-time equivalents, and the 41 PCNs involving some 200 GP practices make up approximately 5% of the overall workforce. There is a significant need, however, for new mental health practitioner roles that can bring benefits to patient care, career development, and progression, extending and securing new routes into health careers, freeing up and generating new capacity and maximizing opportunities in terms of workforce supply routes.

### The challenge

In 2021, the University of Central Lancashire partnered with HEE and the Innovation Agency (North-West Coast Academic Health Science Network) to launch a new NHS role across the North-West Coast system, including in Lancashire and South Cumbria. The Associate Psychological Practitioner (APP) course enables psychology graduates to enter the NHS workforce at increased and higher volumes, thereby producing a sustainable supply of practitioners into psychological roles to reduce significant workforce gaps and change NHS workforce structures. Individuals train for 12 months in the NHS as a Band 4 Trainee Associate Psychological Practitioner, progressing to a Band 5 role as APP upon qualification. In primary care settings, at the time of writing, the role is one of health promotion and prevention, typically delivered as a brief intervention of four sessions to individuals experiencing stress, reduced well-being and/or common mental health symptoms.

The APP course is being rigorously evaluated and this includes an academic and clinical service evaluation which attests to the success and value of the role in a range of services, including PCNs (Budd, *et al.*, 2022; Gardner, *et al.*, 2022). In addition, the project team aimed to model the impact of introducing the APP role into primary care, specifically in terms of generating additional capacity and freeing up GP capacity (appointments). The clinical and project leads worked with the Workforce Repository and Planning Tool (WRaPT) team to develop a project to do that modelling and in turn provide further evidence to underpin decision-making with respect to future cohorts and contribute to wider workforce planning in primary care. We report the approach and findings below.

## Method

Taking a clinically led approach was essential to ensure the validity of any modelling; therefore, the appropriate starting point was to draw upon the learning from two psychology graduates, working in primary care in Lancashire and South Cumbria as part of a HEE feasibility study that aligns well to the clinical activities of the APP role. The WRaPT Team also met with clinical leads and advisors to understand the scope of the work and the kind of output that was needed. This helped identify the type of modelling product to develop and in formulating the assumptions for use in the modelling.

Excel was used to build something that could define future state scenarios and calculate the impact of change determined by the experience and data of two psychology graduates working in a PCN (rather than actual workforce data from a service) to model future state scenarios and the associated change on the baseline workforce. As a starting point, the WRaPT Team generated a set of questions which, once answered, would help to define the assumptions used in the modelling. These included a) identifying the population in scope, b) determining the workforce in scope, c) documenting the activity undertaken by the APP role in terms of volume and the amount of time spent doing it, and d) considering the future state scenarios for modelling.

### Step 1: identifying the patient population in scope

The first stage was to develop an understanding of how many patients the new APP role could potentially see within their scope of practice. The assumption was that the APP role would work with people presenting with mild to moderate mental health needs, who would benefit from a brief psychological intervention.

Those working in the feasibility study were already working with patients of any age however, the scope for the modelling was agreed to restrict the age profile to adults only (those aged 16+). The WRaPT Team used data from NHS Digital to determine the number of patients on practice lists within the PCN where the two psychology graduates worked (NHS Digital, General Practice – Practice Level data, 2022).

Data were extracted for the patient population aged 15+ due to the way it is available via the source. The next step was to refine this information further by identifying the percentage of mental health needs. Different sources were used to inform and direct the lead clinicians to refine the population in scope.

With regard to the prevalence of mental health difficulties, around 19% of adults experience some form of depression, while 17% experience some form of anxiety (approx. 40%; Office of National Statistics, 2020). This also correlates with data on the number of mental health cases dealt with by the GP, which equates to 40% (Mind, 2018). Therefore, we reasoned that up to 40% of the total population of patients within a practice was in scope.

The final stage was to define the severity of the cases as we had already agreed on an assumption that the APP role would deal with mild cases of mental health need as stated above, though some patients entering the service presented with ‘moderate’ need, according to scores on the Patient Health Questionnaire-9 (Kroenke and Spitzer, 2002) and Generalized Anxiety Disorder-7 (Spitzer *et al.*, 2006).

According to NICE Guidelines on depression in adults, mild depression accounts for 70% of all cases and moderate depression 20%, with severe depression accounting for 10% (NICE, 2011). We applied a figure of 70% of the 40% of the ‘in scope’ patient population to determine the number to include in the modelling. For example, if the total patient list size is 10 000, then 4,000 (40%) of these would have mental health difficulties, and of this 4,000, a further 2,800 (70%) would be in the low to moderate range (note that these figures above are for demonstration purposes only and do not relate to the data from the PCN used to inform the project).

### Step 2: determining the workforce in scope

GP workforce data were collated. During this step, we also considered that other roles (e.g., nurses) in a practice might make referrals to the new role of APP; however after consideration, it was concluded that to include them in the modelling more work and data would be required to fully understand how often and when this occurs; hence, it was agreed to exclude them at this time.

The WRaPT Team used data (NHS Digital, General Practice – Practice Level data, 2022) to identify the number of WTE GPs working in the PCN, and who could therefore make referrals to an APP in a new future state, to inform the assumptions. As the APP role did not previously exist in the workforce, it wouldn’t therefore be included in the current state (also known as the baseline model). The modelling would enable future state scenarios to be tested to show the impact of the new APP role on GP appointments, and the APP role would be added as a new source of capacity.

### Step 3: documenting the activity of APPs

At the time of the project, appointment data were available at Clinical Commissioning Group (CCG level) only, rather than at the PCN level which would fit best with the scope of the project. Therefore, to understand the appointments associated with the population of the PCN we were using in the modelling, we had to calculate the PCN population as a proportion of the entire CCG population and then apply that proportion to the total number of appointments in the CCG. The figures used in Table 1 below are from data reflecting the CCG/PCN used for formulating the assumptions and scenarios.

**Table 1.** Data reflecting the CCG/PCN used for formulating the assumptions and scenarios

CCG or PCN	Population figures
CCG Total^*^	397 609
PCN Total^*^	51 198
PCN Aged 15+	39 844
PCN population aged 15+ *(as a proportion of CCG total)*	10%

* NHS Digital Sep-22.

The next step was to apply this percentage to the number of appointments in general practice (GP) to the CCG total. Appointment data can be broken down into ‘GP’ and ‘Other Staff’, and for this purpose, we used GP appointments only (Table 2).

**Table 2.** Total number of GP appointments

CCG/PCN	Number of GP appointments
CCG Total^*^	882 000
PCN Total^*^	88 200

* NHS Digital Oct-21–Sep-22.

*Note:* The number of appointments as a CCG total is for all ages (Table 2), and the data available to us for this work, did not distinguish the numbers of appointments by age group; therefore, we could not identify how many appointments were provided to those aged 15+. Therefore, we decided to use the size of the PCN population (Table 1, all ages) as a proportion of the CCG patient population (which for this model was 10%), this reflects the size of the PCN population to the overall number of appointments. This was the finest level of detail we could model at that time.

These figures gave us an assumption of the total appointments provided for the population in scope for the PCN. The next step was to understand the number of mental health appointments as a proportion of the total.

It has been widely determined that around one in three GP appointments involves mental health (London Strategic Clinical Network for Mental Health, 2014). Therefore, an assumption was applied to the modelling that 33% of the time is spent on Mental Health and 67% of the time is spent on ‘Other’ activity and applying this to the total PCN appointments gives us the number of appointments for Mental Health (Table 3).

**Table 3.** Total number of PCN appointments for mental health

Appointment type	Number of appointments
Appointments – mental health (33%)	29 106
Appointments – Other (67%)	59 904
Appointments – Total	88 200

### Step 4: considering the future state scenarios – Introducing the new APP role into primary care

When looking at the different tasks the new role of APP could carry out, we used the example of the psychology graduates working in the PCN feasibility study as the starting point, which based them on a single practice within the PCN. Using this information and data, we determined that between 3 and 4 days were spent working on ‘cases’, up to 1 day was spent on ‘Community Engagement’ type activities and 2–3 h were spent on ‘Other’ activities which included supervision (data received from clinical lead).

As assigning the activity of mental health appointments (of appropriate patients) to the new APP role was expected to reduce the time GPs spend on mental health appointments, we wanted to model this to quantify the impact of that shift. To model this we used data from a recent review of the impact of social prescribing on healthcare demand in 2017 which showed an average 28% reduction in demand for GP services (Polley and Pilkington, 2017). Taking this into consideration, we applied an assumption that we could reduce the take up of GP appointments (by the population in scope) by between a quarter and one-third.

### Step 5: build the models

First, we had to define what the week of an APP might look like and how their time might be spent. Through discussion with the clinical leads and data from the psychology graduates working as part of the feasibility study, we determined that numerous different combinations of activity could build up to make a week of work. For the modelling, we assumed 45 weeks per year of availability to allow for annual leave and other time off and 37.5 h per week of available hours to work. The types of activity that the APP could do were then categorized as follows:

#### Community engagement

- Well-being Promotion (via Community Engagement) would account for half a day per week. The patients that the APP would engage with under this activity would be all mild cases that are not on the caseload.

#### Other/supervision/mentoring

- Other (including supervision and mentoring) would be for around a quarter of a day per week.

#### Group work

- Group work would offer a weekly half-day session for 8 weeks. Following this, the APP would do community well-being sessions for four weeks.
- These sessions would be for half a day a week and up to 12 participants.
- Assuming 45 weeks of activity there could be three full cycles (both group work and well-being) and one cycle of group work.
- The APP delivers the group sessions jointly with another general practice colleague (e.g., a social prescriber) however the modelling reflects only the APP time as other primary care staff are out of scope for this project.

#### Brief interventions

- Brief intervention appointments with patients in scope – the APP would do these for 3–4 days a week (depending on other activity undertaken) and they would take 45 min per appointment.

#### Triage sessions

- By introducing the APP role, a possible scenario is that APPs would be a first-contact clinician and see the patient if they requested an appointment relating to mental health need, for an assessment (also referred to as a triaging appointment). These appointments would be 30 min long.

Some of the activities are based on a fixed amount of time or volume and others could be more or less volume depending on how the week is structured. Using the activities, whether they were fixed or variable, and the time taken, we identified three different types of week (Table 4). The standard week 1 involves three main types of activity, whereas alternative weeks 1 and 2 include additional clinical activities.

**Table 4.**
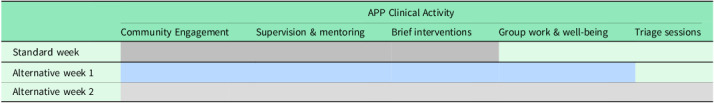
Three alternative APP working weeks differentiated by clinical activity

## Findings

We built the APP standard week and alternative weeks so we could model different scenarios by:

1. Introducing the new APP role at 1.0 WTE.
2. Adding combinations of activities that the APP could carry out to create numerous different scenarios.
3. Applying the findings from the above to understand the potential impact on GP appointments by having the APP role in primary care working with patients identified as in scope.

One scenario could be for the GP to assess the patient in one appointment and then refer to the APP for a series of brief psychological interventions to meet their mental health need. As the APP would be seeing mild-moderate cases, for this scenario we estimate that these patients might have seen the GP three or four times depending on whether they experience physical health complaints, mental health complaints or, both. Those presenting with both are at an increased likelihood of presenting to the GP more frequently (Saini *et al.*, 2020).

### Generating additional capacity

Assuming 45 weeks of activity there could be three full cycles (both group work and well-being) and one cycle of group work; hence up to 48 people (assuming 12 participants in each of the 4 cycles) could benefit from attending Group and Well-being sessions over a year, totalling 384 intervention sessions.

### Number of GP appointments diverted per PCN (based on the standard week of APP clinical activity)

For this scenario, the future state accounts for the change in flow (i.e., the number of GP appointments before seeing an APP) and we have modelled the impact of such by reducing the number of appointments with the GP for the group of patients in scope by both three and four appointments to account for the possible number of presentations as outlined above. For example, based on the range of activities being undertaken by the APP (which we have calculated but is variable), the modelling shows that 1,665 appointments could be offered with the APP that ordinarily might have been undertaken by the GP. This would be based on having 1 WTE APP across a PCN or within a number of practices, undertaking a variety of activities. We can use this to calculate that between 13 650 and 15 280 GP appointments could be freed up by introducing this change in flow to all the patients in scope in the PCN and employing the appropriate number of APPs (8.2 and 9.2 WTE respectively). We have also used this to calculate the APP activity that could be realized by having 1 in each PCN in Lancashire and South Cumbria.

### Number of GP appointments diverted per PCN (based on alternative week 2 of APP clinical activity) and across all 41 PCNs

The detailed model in Table 5 shows the potential impact of changing the flow of activity from GP to APP based on alternative week 2 as a representative example of an APP’s annual clinical activity and a current state of three appointments with the GP, moving to a future state of one appointment with the GP who refers the patient to the APP for a follow-up session. Note that the results will vary as a function of APP clinical activity, e.g., the use of the standard week, or alternative week 1 or 2. Based on this model, we also calculated the number of GP appointments diverted across all 41 PCNs. Table 5 shows that 53 000 GP appointments could be diverted per annum to an APP, if APPs are offering brief intervention appointments of 45 min each and with one APP in each of the 41 PCNs.

**Table 5.** Number of appointments with 1 × APP in each of the 41 PCNs (GP appointments diverted)

GP appointment flow	Number of GP mental health appointments at the PCN level
GP appointments for mental health total	29 106
70% appointments in scope for APP	20 374
1/3 remain with GP	6,723
2/3 move to APP	13 650
Appointments with 1 × APP in each PCN (GP appointments diverted)	
GP appointments diverted – based on APP activity in Alternative Week #2 and 1 × APP in each PCN (41)	>53 000

Another scenario is that of patients having an alternative referral route to the APP including self-referral or via another practitioner or other service. This would mean they don’t see the GP at all, and the APP undertakes a first-contact triage appointment. Whilst we could apply any percentage here for alternative referral, we felt that as the patient would be bypassing the GP entirely it was appropriate to model this in the first instance as being around 10% of patients fitting within the scope for this new route. That is, our assumption modelled 10% of patients self-referring to an APP (further data gathering over time could lead to refined modelling of the impact on the GP using a larger % of self-referrals to an APP, if appropriate). Table 6 shows that if just 10% of patients within just 1 PCN self-referred to an APP, this would result in 2,038 APP appointments per year for that PCN, leading to less demand on GPs. However, the activities undertaken by the APP would determine how many of these 2,038 appointments could be absorbed by 1 WTE APP.

**Table 6.** Number of self-referred appointments with 1 APP per PCN per annum

Appointment flow	Split of mental health appointments with GP or self-referred to APP
GP appointments for mental health total	29 106
70% appointments in scope for APP	20 374
9/10 appointments remain with GP	18 336
1/10 appointments directly self-refer to APP	2,038

## Discussion

Working with the WRaPT Team and their method of formulating assumptions based on data and findings of a feasibility study enabled us to evaluate how the APP workforce can help increase the capacity of General Practice colleagues and meet mental health needs. Given the pressures on the workforce in this setting and the rising need, this work is both important and timely. Our first key finding was that APPs provide additional mental health workforce capacity. Having 1 APP in each of the 41 PCNs in Lancashire and South Cumbria could provide 53 000 brief intervention appointments of 45 min each, thereby diverting these appointments away from the GP; and up to 48 people could benefit from attending Group and Well-being sessions over a year with 1 APP working with another Primary Care colleague, that is, 384 group intervention sessions delivered. Second, we found that APPs can free up GP capacity by seeing clients with mental health needs. One APP (if placed across a PCN or within a number of practices over the 41 PCNs) could free up at least 1,665 GP appointments within one year.

### Limitations, implications and future work

Working with the WRaPT team and following the method described above has allowed us to quantify the potential impact of APPs in Primary Care by producing immediate realistic estimates of the number of GP appointments that could be diverted if just 1 APP were to be placed in 41 PCNs. This would in turn generate significant NHS cost savings. Our recommendations are preliminary as they are based on modelling of *future* state scenarios, that is, the potential number of GP appointments that ‘could’ be diverted if we expand the current workforce of APPs into more PCNs. Future work will need to use patient data collected by APPs to facilitate a full analysis of cost-effectiveness, e.g., client scores on routine clinical outcome measures such as the EuroQol five-dimension questionnaire (EuroQoL Research Foundation). Indeed, future work that quantifies NHS cost savings is an important endeavour. We recommend that with the appropriate input and engagement from clinical leads and access to data, the impact of introducing the APP role more widely should be modelled and evaluated, e.g., within different care pathways. Strong clinical engagement is essential to ensure that the scope of practice for the APP role is based on sound rationale and safety. Indeed, clinical expertise was essential in informing the data collection and refining of the modelling throughout the project. The method devised for the APP modelling could be used for other new roles/in different settings with the appropriate engagement and data. Whilst we determined the potential impact of introducing the APP role into primary care, we also developed a translatable method.

It is important to understand that our work involved making assumptions about the patient population/workforce in scope, the nature of the clinical activity undertaken by APPs, and the potential number of patients that might self-refer to an APP, and that as a result, our estimates of APP capacity and GP capacity will vary as a function of model parameters. For example, we described three alternative weeks to demonstrate different types of clinical activity that APPs might engage in, yet these are simply three possible working week scenarios and there may be other clinical activities that APPs engage in. Similarly, our modelling was based on key assumptions about these clinical activities, such as up to 12 people attending a group work session, though the actual number attending could vary each week.

A key strength of this work is that our tool was designed to be a flexible model that could be adapted (e.g., based on APP clinical activity and for different pathways), facilitating replication and adaptation of our estimates. As further cohorts of APPs move through their training and into posts, model assumptions can be reviewed to ensure they remain realistic. Using further data to build up strong assumptions is key to the ongoing validation of the modelling assumptions.

Regarding the transferability of the model to other primary care settings, we expect this will depend on the maturity of the PCN in terms of infrastructure, resources, and relationships. This pilot took place in a well-established PCN environment with general practice engagement. This informed the modelling as it helped to determine the scope of practice and support needed for the APP role to provide care successfully and safely for appropriate patients and the subsequent impact on the GP. In less mature or established environments this might not be so successful. The APP role could be deployed within a PCN environment, within a single practice or in other settings. The WTE proportion of the APP role required, the volume of cases seen, and the impact therefore on GPs would be determined by adjusting the model using relevant data.

## Conclusion

This is the first paper to determine the potential impact of introducing APPs into Primary Care settings in terms of both the additional capacity to provide mental health care and the impact on GP capacity within the sector, which could generate NHS cost savings. These findings can be used to underpin decision-making with respect to future cohorts and contribute to wider workforce planning in primary care.
